# The effect of nutrition and body condition of triplet-bearing ewes during late pregnancy on the behaviour of ewes and lambs

**DOI:** 10.5713/ajas.17.0890

**Published:** 2018-04-12

**Authors:** Gabriella V. Gronqvist, René A. Corner-Thomas, Paul R. Kenyon, Kevin J. Stafford, Stephen T. Morris, Rebecca E. Hickson

**Affiliations:** 1 International Sheep Research Centre, Institute of Veterinary, Animal and Biomedical Sciences, Massey University, Palmerston North, 4442, New Zealand

**Keywords:** Triplet Lambs, Late Pregnancy Nutrition, Body Condition Score, Ewe and Lamb Behaviour, Vocalisation

## Abstract

**Objective:**

Triplet-born lambs are less likely to survive to weaning than twin-born or single-born lambs. Appropriate ewe-lamb bonding behaviours and lamb vigour behaviours are necessary for survival of lambs. The aim of this experiment was to determine whether maternal nutrition during late pregnancy influenced behaviour of the ewe and her lambs soon after birth, and to determine whether mid-pregnancy body condition score (BCS) influenced any behavioural response.

**Methods:**

The experiments included ewes that were in BCS 2.0, 2.5, or 3.0 in mid-pregnancy and were fed either *ad libitum* or to pregnancy-maintenance requirements in late-pregnancy (day 115 until 136 in experiment one, and day 128 until 141 in experiment two). The time taken for lambs to stand, contact dam, suck from dam and follow dam was recorded three to 18 h after birth. The number of high- and low-pitched bleats emitted by the ewe and lambs was recorded, along with maternal behaviour score (MBS) of the ewe. Lambs in experiment two underwent a maternal-recognition test at 12 or 24 h.

**Results:**

There were significant effects of feeding treatment on bleating behaviour of ewes and lambs, but these were inconsistent among BCS groups and between experiments. Lamb vigour behaviours were not affected by feeding treatment. In experiment one, there was no effect of feeding treatment or BCS on MBS, but in experiment two, ewes in BCS3 in mid-pregnancy had greater MBS than ewes in BCS2 in mid-pregnancy (MBS 3.1/5 vs MBS 2.1/5; p<0.05).

**Conclusion:**

Given there were no repeatable effects on behaviour of ewes and lambs, *ad libitum* feeding rather than feeding for pregnancy-maintenance requirements cannot be used to improve behaviours soon after birth of triplet-bearing ewes in BCS 2–3 and their lambs in extensive pastoral conditions.

## INTRODUCTION

Advances in sheep production in New Zealand have increased the lambing percentage (lambs weaned per ewe joined) from 98% in 1987 to 127% in 2017 [1]. This is associated with a fecundity rate (number of fetuses per ewe) in the range of 140% to 170%, and has resulted in a corresponding increase in the number of triplet-born lambs [2], which are less likely to survive to weaning than single-born or twin-born lambs [2]. Triplet-bearing ewes make up around 10% of the ewe flock, and the death rate of triplet-born lambs is 25% to 35% [2,3]. Lamb deaths are a concern to the sheep industry from both an animal welfare and a financial perspective [4].

In New Zealand’s spring-lambing system, feed demand is greater than pasture growth rate in winter, and as a result, feeding levels of ewes are controlled. The general aim is to ensure that ewes are fed at pregnancy maintenance level, such that total liveweight gain is equivalent to the expected increase in conceptus weight [5]. For triplet-bearing ewes on a pregnancy maintenance level, the conceptus weight at full term ranges between 18 to 21 kg [6], and much of this weight is gained in the last three weeks of gestation, when the conceptus weight increases at a rate of 450 g/d for triplet litters [5].

Morris and Kenyon [7] and Kenyon et al [8,9] reported that there was no increase in birth weight or pre-weaning growth rates of triplet lambs when ewes were offered unrestricted (*ad libitum*) pasture allowances compared with pregnancy maintenance feeding levels in late pregnancy. Further, these experiments revealed that there is little benefit in terms of lamb live weight from having a ewe body condition score (BCS) greater than 3 in late pregnancy [8–10], and ewes in New Zealand are typically maintained in BCS 2–3 during pregnancy [10]. There is a lack of knowledge, however, regarding the effect of both feeding and BCS of ewes in late pregnancy on behaviour of triplet-born lambs. Vonnahme et al [11] reported endocrine changes in ewes fed above pregnancy maintenance, even when differences in live weight were not detected, so it is possible that there may be hormonally mediated changes in behaviour of ewes and lambs in response to changes in feeding level. Lamb and ewe bleating behaviour may be used as an indication of the strength of the ewe-lamb bond [12,13]. High-pitched bleats are often emitted when the ewe and lambs are separated and may indicate a degree of need [14]. Gronqvist et al [15] reported that twin-bearing ewes on *ad libitum* feeding allowance in mid pregnancy expressed fewer high bleats at handling of their lambs within 18 h of birth than those that had been on pregnancy maintenance feeding level in mid pregnancy. Additionally, their lambs differed in number of high and low pitched bleats expressed. Low-pitched bleats help strengthen the ewe-lamb bond and are also considered ‘care-giver’ bleats emitted exclusively between the ewe and her lamb [12,13,16]. Corner et al [17] reported that twin- and triplet-bearing ewes that had grazed on 4 cm pasture compared with 2 cm pasture from day 108 of pregnancy until lambing emitted more low-pitched bleats, although there was no effect on lamb bleating behaviour in that study.

A strong ewe-lamb bond is necessary to facilitate suckling and ongoing care of the lamb, and appropriate behaviours including grooming, standing, sucking and bleating by ewes and lambs in the first few hours after birth are needed to develop this bond [18]. Studies of single and twin-born lambs have reported differences in the behaviour of ewes and newborn lambs in response to changes in pregnancy nutrition. Previous studies examining the effect of undernutrition (less than pregnancy maintenance) have shown negative impacts on both maternal [19–21] and lamb behaviour [21,22]. Gronqvist et al [15] reported that *ad libitum* feeding of twin-bearing ewes during late pregnancy did not improve the behaviour of the ewe or her newborn lambs. However, given that the nutritional demands on triplet-bearing ewes are considerably greater than twin-bearing ewes [23], there may be greater scope for feeding level and body condition to influence behaviour of triplet-bearing ewes and their lambs. Therefore, the aim of these experiments was to determine whether body condition in mid-pregnancy and feeding level under pastoral conditions during late pregnancy of triplet-bearing ewes influenced behaviour of the ewe and her lambs.

## MATERIALS AND METHODS

Two experiments were conducted at Massey University’s Keeble farm, 5 km south of Palmerston North, New Zealand. The experiments were conducted with approval from the Massey University Animal Ethics Committee. Observing ewe and lamb behaviour immediately after birth is not practical in an extensive grazing situation, where ewes may flee on approach of the observer prior to formation of an adequate bond. Therefore, observation of behaviour is frequently made a few hours after birth, after bonding is established, in these situations [17, 19,21]. The experiments, measurements and analyses described here follow the same procedures as Gronqvist et al [15].

### Animals and treatments

#### Experiment one

This experiment included 119 Romney ewes aged 3 to 5 years of age and their 357 lambs, these were a subset of 148 ewes used by Kenyon et al [8]. Only complete sets of triplet-born lambs (all lambs alive at time of tagging) and their dams were included in the experiment. Ewes were identified as triplet-bearing at pregnancy scanning 72 days after the start of the breeding period (D72) and were in BCS 2, 2.5, or 3 at D72 after having been managed as a single group [8]. At D115, ewes from each BCS group (BCS2, BCS2.5, BCS3) were allocated to either pregnancy maintenance feeding (“medium”) or *ad libitum* feeding on ryegrass-dominant pastures with <5% white clover for 22 days, until D136, nine days prior to the expected start of lambing. During P115 to P136, the pre-grazing herbage metabolisable energy (ME) content of the pasture offered to the medium and *ad libitum* groups were 14.0±0.4 MJ ME/kg dry matter (DM) and 13.1±0.4 MJ ME/kg DM, respectively [8]. From D136, all ewes were grazed in a single group and offered *ad libitum* feeding until lambing. Pregnancy maintenance feeding was achieved with mean (±standard error of the mean [SEM]) pre- and post-grazing pasture masses of 1,578±47 kg DM/ha and 895±37 kg DM/ha, respectively. *Ad libitum* feeding was achieved with mean (±SEM) pre- and post-grazing pasture masses of 1,973±47 kg DM/ha and 1,210±35 kg DM/ha, respectively. A post-grazing herbage mass above 1,200 kg DM/ha on ryegrass/white clover pasture is sufficient to allow unrestricted intake for ewes [7]. Ewes entered lambing paddocks on D141, onto pre-grazing pasture mass of 1,577±31 kg DM/ha, and during lactation mean pasture masses was 1,295±26 kg DM/ha. All ewes lambed over a 20-day period.

#### Experiment two

This experiment included 88 Romney ewes aged 3–5 years of age and their 264 lambs. These were a subset of the ewes used by [9]. Only complete sets of triplet-born lambs (all lambs alive at time of tagging) and their dams were included in the experiment. Ewes were identified as triplet-bearing at pregnancy scanning 72 days after the start of the breeding period (D72) and were in BCS 2, 2.5, or 3 at D72 after having been managed as a single group [9]. At D128, ewes within each BCS group (BCS2, BCS2.5, BCS3) were allocated to either pregnancy maintenance feeding (medium) or *ad libitum* feeding on ryegrass-dominant pastures with <5% white clover for 14 days, until D142, 4 days prior to the expected start of lambing. Pasture ME was not measured, however, ryegrass dominant swords in spring are associated with ME of 10.4 MJ/kg DM and a crude protein content of 24.7% to 26.3% [24]. From D142, all ewes were grazed in a single group and offered *ad libitum* feeding until lambing. Pregnancy maintenance feeding was achieved with mean (±SEM) pre- and post-grazing pasture masses of 1,125±58.5 kg DM/ha and 807.9±58.5 kg DM/ha, respectively. *Ad libitum* feeding was achieved with mean (±SEM) pre- and post-grazing pasture masses of 1,744±64.1 kg DM/ha and 1,254±61.1 kg DM/ha, respectively. Ewes entered lambing paddocks on D142, onto pre-grazing pasture mass of 1,254±61.1 kg DM/ha, and during lactation mean pasture masses was 1,574±43.8 kg DM/ha. All ewes lambed over a 19-day period.

Experiment two considered a shorter period of *ad libitum* feeding because this was more likely to be achievable on-farm during late pregnancy in late winter when feed is often in short supply. The treatment period was also moved as late as possible into pregnancy so as to occur as close as possible to lambing as would be practical to achieve on farm. Under extensive pastoral conditions, ewes must be set-stocked into lambing paddocks at least a few days prior to the expected start of lambing, because moving ewes and lambs during lambing contributes to mis-mothering of lambs.

### Measurements

Live weight and BCS of the ewes in the two experiments were recorded at D90, D115, D136, and D142 and D83, D114, D128, and D142, respectively (BCS on the 1–5 scale [10,25]). During the lambing period, ewes were inspected twice daily at 8 am and 4 pm. Lambs were handled once their coat was dry and all lambs in the litter were mobile (at approximately 3 to 18 h of age). During handling, lambs were identified to their dam, ear-tagged and had their weight, birth-rank and sex recorded. Immediately following handling, the three lambs were placed together, lying on the ground while three observers moved approximately 10 metres away. The moment the lambs were released was considered to be ‘time zero’. The observers recorded the behaviours of the individual lambs and the ewe during the next five minutes. These observations were made on all ewes and lambs in experiment one, and on 71 ewes and their 213 lambs in experiment two.

The behaviours recorded included the time at which the lamb stood (defined as fully supporting itself on all four legs for at least five seconds), the time at which the lamb and ewe made contact (defined as being within 0.5 metres of each other [15,21,26]), the time at which the lamb followed the ewe at least 5 m from their first point of contact [15,21], and the time at which the lamb successfully sucked from its dam’s teat (lamb held teat in its mouth and appeared to be sucking for at least five seconds; [15]). These variables were recorded as the number of seconds from the start of observation until the behaviour was observed. Lambs that did not display the behaviour were given a value of 301 s.

The observers also counted the total number of low-pitched bleats (bleats involving little mouth movement) and high-pitched bleats (bleats involving full mouth movement) emitted by each lamb and by the ewe [15,21]. Maternal behaviour score (MBS) was assessed on a five-point scale [27] for each ewe based on the distance the ewe moved away from her lambs while the lambs were being handled (one = at the approach of the shepherd ewe flees and does not return, two = ewe moves away >10 m but returns to lambs as shepherd leaves them, three = ewe retreats 5 to 10 m from lambs, four = ewe retreats but stays within 5 m of her lambs, five = ewe stays within 1 m and makes contact with the shepherd and lambs).

#### Maternal-recognition test

**Figure 1:**
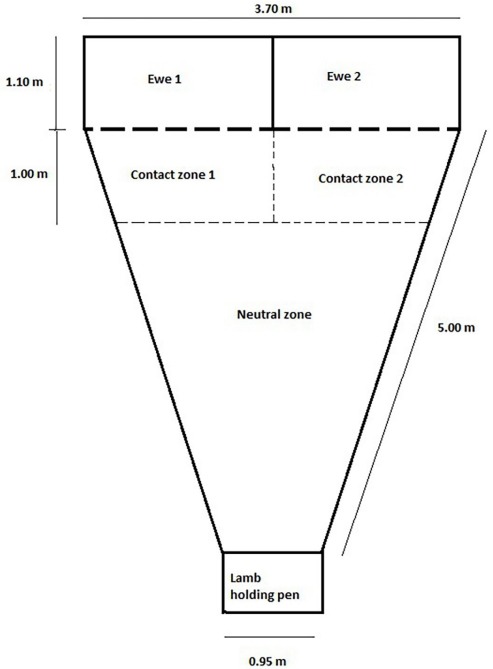
Diagram showing layout of the testing arena and holding pens for the maternal-recognition test (adapted from Nowak et al [28]).

In experiment two only, all lambs were subjected to a maternal-recognition test [15], similar to that described by [28,29]. The lamb’s ability to discriminate its dam has been used as an indicator of ewe-lamb bond strength [28,29], an aspect of ewe and lamb behaviour that could not be measured through paddock observations. The testing arena was of a triangular shape fenced by one metre high solid walls (3.7×6.1 m, Figure 1). Adjacent to the vertex of the triangle pen was a lamb holding pen. At the base end of the triangle arena were two pens (1.85×1.1 m) side by side, separated from the testing arena by wire-mesh gates. The arena itself was divided into three zones separated by lines drawn on the ground; a neutral zone (the area of the triangle that was more than one metre from either ewe pen), and two ewe contact zones adjacent to the ewe pens.

Testing occurred between 1 pm and 3 pm daily. As a result, lambs were tested at approximately 12 (n = 168) or 24 (n = 96) h of age depending on when they were born and handled. The dam of the lamb being tested was placed randomly in one of the two ewe pens, with an ‘alien’ ewe which had lambed at a similar time placed in the other pen. Each lamb was tested individually and was placed, standing, in the lamb holding pen facing the two ewes. Once the lamb was released into the neutral zone the lamb could see both ewes in the pens. All other lambs, including the sibling of the lamb being tested and the lambs of the alien ewe were kept approximately five metres away so that the ewes could hear them but not see them. Each lamb was only tested once to avoid any possible learning effects [29,30].

Each maternal-recognition test was conducted for five minutes and the location and activity of the lamb were recorded at 10 s intervals. This allowed the total time that the lamb spent in the contact zone adjacent to its dam, or in either contact zone, and the time spent sitting and walking to be calculated. High and low-pitched bleats were counted for the duration of the test.

### Statistical analysis

Statistical analyses were conducted using SAS v9.3 (SAS Institute Inc., Cary, NC, USA). Each experiment was analysed separately.

Live weight and BCS of the ewes were analysed using a mixed model that allowed for repeated measures and included the fixed effects of ewe nutrition treatment and BCS group and their interaction, the covariate “days to lambing”, which was the number of days between D140 and the lambing date of the ewe, and the random effect of ewe.

The percentage of lambs or ewes that performed each behaviour (bleating, paddock behaviours, or entering a specific zone in the triangle-pen test) was analysed using generalised models based on a binomial distribution and a logit transformation. These models included the fixed effects of feeding treatment and BCS group, and their interaction when it was significant (p<0.05). For the maternal-recognition test behaviours, age of the lamb at testing (approximately 12 or 24 h) and all significant (p<0.05) two-way interactions among age, feeding treatment and BCS group were also included in the model. Results are presented for the interactions where they were significant.

The number of bleats emitted was tested for normality using the Kolmogorov-Smirnov test. It was not normally distributed and could not be normalised. Number of bleats emitted by animals that emitted at least one bleat was analysed using a generalised model based on a Poisson distribution and a log transformation. Lambs or ewes that did not express at least one bleat of the type considered in the model were excluded from the analysis of number of bleats. The models included the fixed effects of feeding treatment, BCS group and their two-way interaction when it was significant (p<0.05). Lamb bleating behaviour in the maternal-recognition test also included the fixed effects of age of lamb at testing (approximately 12 or 24 h) and the interaction of ewe feeding treatment with age of lamb and ewe BCS with age of lamb if these were significant (p<0.05). Analysis of number of bleats included only those ewes or lambs that emitted the bleat of interest at least once.

The time required for lambs to stand, make contact with their dam, suck from their dam and follow her when she moved away during the paddock observations after handling was tested for normality using the Kolmogorov-Smirnov test. All data were not normally distributed and could not be normalised, even when lambs that did not show the specific behaviour were excluded. The median time to exhibit each behaviour was calculated using the non-parametric Kruskal-Wallis test. The effects of ewe feeding treatment and BCS group were tested in separate models and the analysis contained all lambs including those that did not show the behaviour during the observation period. The association between feeding treatment or BCS group and the behaviour variable was investigated using the Wilcoxon test. For variables for which BCS group had a significant effect (p<0.05), the Wilcoxon two-sample post hoc test with a Bonferroni adjustment was carried out to determine differences among groups. Time spent walking, sitting, in the contact zone or with the dam during the triangle-pen test were also not normally distributed and were analysed using the same approach, with the addition of age of lamb at testing to the effects tested.

Ewe MBS was analysed using a generalised model assuming a Poisson distribution and a log transformation. The model included the fixed effects of ewe feeding treatment and BCS group. The interaction between feeding treatment and BCS group was considered but removed because it was not significant (p>0.05).

## RESULTS

### The effect of ewe feeding treatment and BCS group on ewe live weight and BCS

In experiment one, ewes in the *ad libitum* treatment had greater (p<0.05) BCS than the ewes in the medium treatment at the end of the feeding period (D136), but live weight was similar (p>0.05; Table 1). In contrast, in experiment two, ewes in the *ad libitum* treatment were heavier (p<0.05) but not different (p>0.05) in BCS to ewes in the medium treatment. In experiment one, differences in BCS that existed at D72 were no longer present by D136, whereas in experiment two, ewes that were BCS3 at D72 remained in greater BCS throughout the experiment than ewes that were BCS2 at D72. There were no significant interactions between treatment and BCS group.

### Vocalisation of lambs after handling

In experiment one, there was no difference between treatments or among BCS groups in the percentage of lambs that expressed high or low bleats (p>0.05; Table 2). In contrast, in experiment two, a greater percentage of lambs born to BCS3 ewes expressed high and low bleats, compared with lambs born to BCS2 ewes (p<0.05). Furthermore, a greater percentage of lambs born to ewes in the medium treatment expressed high bleats than did lambs born to ewes in the *ad libitum* treatment (p<0.05).

In both experiments, there was an interaction between feeding treatment and BCS group for both number of high bleats and number of low bleats. Among lambs born to ewes in the *ad libitum* treatment, those from ewes in the BCS3 group expressed fewer (p<0.05) high bleats in both experiments, whereas among lambs born to ewes in the medium treatment, those from ewes in the BCS3 group expressed the least (p< 0.05) high bleats in experiment one and the most (p<0.05) high bleats in experiment two. Lambs born to BCS2 ewes in the medium treatment expressed more low bleats than those born to BCS2 ewes that in the *ad libitum* treatment (p<0.05), but there was no difference between feeding treatments for the BCS2.5 group in both experiments (p>0.05). Lambs born to BCS3 ewes expressed more low bleats in the *ad libitum* compared with medium treatment in experiment one (p<0.05), but a similar number of bleats between treatments in experiment two (p>0.05).

### Vigour behaviour of lambs after handling

In experiment one, the time for lambs to stand, make contact, suck and follow was not affected (p>0.05) by the feeding treatment or BCS group of the ewe (p>0.05; data not shown). Similarly, in experiment two, there was no effect of feeding treatment on time for lambs to stand, make contact, suck or follow (p>0.05). Time to make contact or suck were not affected by BCS group in experiment two (p>0.05), however, time to stand and follow differed among BCS groups (p<0.05). Median time to stand was 24.0 s for lambs born to ewes in the BCS2 group vs 16.0 s for lambs born to ewes in the BCS2.5 group (p<0.05). Lambs born to ewes in the BCS3 group had a median time to stand of 23.5 s, which did not differ from those born to ewes in the BCS2 or BCS2.5 groups (p>0.05). Time to follow also differed among BCS groups, in that lambs born to ewes in the BCS 2.5 group were slower (p<0.05) to follow than lambs born to ewes in the BCS2 or BCS3 groups (median time 301.0 s vs 210.0 s and 231.5 s, respectively).

### Behaviour of ewes at handling of their lambs

The MBS of the ewe did not differ between feeding treatments in either experiment (p>0.05, data not shown). The MBS was similar among BCS groups in experiment one (p>0.05; data not shown), but in experiment two, ewes in the BCS3 group had greater MBS than ewes in the BCS2 group (3.1 [95% confidence interval 2.4 to 3.9] vs 2.1 [95% CI 1.6 to 2.8]; p<0.05), whilst ewes in the BCS2.5 group did not differ from either group (2.7 [95% CI 2.1 to 3.4]; p>0.05).

The percentage of ewes that expressed at least one high or low bleat did not differ among feeding treatments or BCS groups in either experiment (p>0.05; data not shown). There was an interaction between feeding treatment and BCS group for number of high bleats and number of low bleats in both experiments (p<0.05, Table 3). Within BCS2 ewes, those in the medium treatment expressed more high bleats than those in the *ad libitum* treatment in experiment one, but fewer in experiment two. *Ad libitum* feeding decreased number of high bleats expressed by BCS2.5 ewes in experiment one but had no effect in experiment two, whereas within BCS3 ewes, *ad libitum* feeding had no effect in experiment one but increased number of high bleats expressed relative to ewes fed pregnancy maintenance in experiment two. The effect of *ad libitum* versus medium feeding on number of low bleats expressed also varied among BCS groups and experiments (Table 3).

### Behaviour of lambs in the maternal recognition test (experiment two only)

The outcome of the maternal recognition test was not affected by ewe feeding treatment, BCS group or age of lamb (p>0.05). Overall, 52% of lambs preferred to spend time with their own dam, 20% preferred the alien dam and 28% displayed no preference between the dams. Similarly, the percentage of lambs that spent time with their dam, with the alien dam or in the contact zone was not affected by feeding treatment or age of lamb, and the percentage of lambs that spent time with the alien dam or in the contact zone was not affected by BCS group (p>0.05; data not shown). The percentage of lambs that spent time with their dam was greater for lambs born to BCS2 ewes compared with those born to BCS3 ewes (97.9% [95% CI 91.9 to 99.5] vs 90.4% [95% CI 82.3 to 95.1], p<0.05), whilst lambs born to BCS2.5 ewes did not differ to either group (94.7% [95% CI 87.6 to 97.9], p>0.05).

A greater percentage of lambs born to ewes in the medium feeding treatment than the *ad libitum* feeding treatment emitted low-pitched bleats during the maternal recognition test (72.9 [95% CI 64.7 to 79.8] vs 55.6 (95% CI 46.9 to 63.9), p<0.05). There were no other effects of ewe feeding treatment, BCS or age of lamb on the percentage of lambs that emitted low- and high-pitched bleats (p>0.05; data not shown).

Of those lambs that bleated, there was a significant interaction of ewe feeding treatment and BCS on the number of high-pitched bleats such that within the BCS 3.0 group lambs born to ewes in the medium treatment emitted more high-pitched bleats than the *ad libitum* treatment (p<0.05, Table 4), whilst the opposite effect was observed within the BCS 2.0 and 2.5 groups (p<0.05). Lambs born to ewes within the BCS 2.0 and 2.5 groups expressed more low bleats in the medium treatment than in the *ad libitum* lambs born to ewes in the medium treatment expressed more bleats than those born to ewes in the treatment (p<0.05), whilst there were no differences within the BCS 3.0 group (p>0.05).

There was also a significant interaction of ewe feeding treatment and age of lamb on the number of high-pitched bleats such that at 12 h of age, lambs from both feeding treatments expressed a similar number of high-pitched bleats (p>0.05), whereas at 24 h of age, lambs born to ewes in the *ad libitum* treatment expressed more high-pitched bleats than those born to ewes in the medium treatment (p<0.05). Conversely for low-pitched bleats, lambs born to ewes in the medium treatment expressed more bleats than those born to ewes in the *ad libitum* treatment at 12 h of age (p<0.05) but number of bleats expressed was similar at 24 h of age (p>0.05).

Ewe BCS group interacted with age also, such that at 12 h of age, lambs born to BCS3 ewes expressed more high-pitched bleats than did lambs born to BCS2 or BCS2.5 ewes (p<0.05), whereas at 24 h of age, lambs born to both BCS3 and BCS2.5 ewes expressed more high-pitched bleats than did lambs born to BCS2 ewes (p<0.05). Conversely, at 12 h of age, lambs born to BCS2 ewes expressed more low-pitched bleats than did lambs born to BCS2.5 or BCS3 ewes (p<0.05). At 24 h of age, lambs born to BCS2.5 ewes bleated least, and lambs born to BCS3 ewes bleated most (p<0.05).

There was no effect of feeding treatment or BCS group on the percentage of lambs that walked or sat, or the amount of time lambs spent walking, sitting or standing (p>0.05, data not shown). Similarly, there was no effect of feeding treatment or BCS group on the time taken for the lambs to reach the contact zone or to reach their dam (p>0.05, data not shown). Lambs tested at 24 h of age spent more time walking (median 70 vs 50 s) and less time standing (median 240 vs 220 s) than lambs tested at 12 h (p<0.05). There was no effect of age on time spent sitting (p>0.05, data not shown). Lambs tested at 24 h took a median of 10 s to reach the contact zone, whilst lambs tested at 12 h took 20 s to reach the contact zone (p<0.05). There was no difference in the time to reach their dam between 12 h and 24 h old lambs (p>0.05, data not shown).

## DISCUSSION

The aim of these two experiments was to investigate whether *ad libitum* compared with feeding to pregnancy-maintenance requirements in late pregnancy had positive effects on the behaviour of ewes and triplet-born lambs soon after birth. The pre- and post-grazing masses in both experiments indicate that nutritional conditions of the feeding treatments differed and met the objectives. The potential impact of ewe BCS over the range of 2 to 3 in mid-pregnancy under these feeding conditions was also examined.

High-pitched bleats are considered distress bleats, and are most frequently expressed when ewes and lambs are separated from each other [14,16]. *Ad libitum* feeding reduced the number of high bleats expressed by BCS2 and BCS2.5 ewes in experiment one, but not in experiment two, when BCS2 ewes in the *ad libitum* treatment expressed more high bleats than those in the medium treatment. Gronqvist et al [15] reported that *ad libitum* feeding compared with feeding for pregnancy maintenance for twin-bearing ewes in late pregnancy resulted in fewer ewes expressing high-pitched bleats. It is unclear why the results of experiment two differ from experiment one and Gronqvist et al [15]. There were no effects of feeding treatment or BCS group on time to make contact with lambs, so these differences do not reflect the time spent separated from their lambs. The results of experiment one and Gronqvist et al [15] indicate that better feeding in late pregnancy results in the ewe being less distressed when separated from her lamb, however, this finding is not supported by the results of experiment one, or by the results of Everett-Hincks et al [21] or Corner et al [17], who both reported no effect of *ad libitum* versus restricted feeding on high-bleating behaviour of ewes.

The MBS was similar among feeding treatments and BCS groups in experiment one, and between feeding treatments in experiment two, similar to the findings of Everett-Hincks et al [21], Corner et al [17] and Gronqvist et al [15] in both twin- and triplet-bearing ewes. In experiment two, ewes in the BCS3 group had better maternal behaviour than ewes in the BCS2 group. Interestingly, this was not reflected in a shorter time to contact their lambs once they were released. Combined, these results indicate that there is no advantage in terms of MBS from feeding triplet-bearing ewes above pregnancy maintenance levels in late pregnancy. Furthermore, there is little, if any, difference among ewes in different BCS in mid-pregnancy, over the range of BCS 2–3.

Vocalisation by lambs in both the paddock and the maternal-recognition test showed inconsistent differences among treatments and BCS groups. This is similar to the findings of Gronqvist et al [15] for twin-bearing ewes. Lamb bleating behaviour is affected by both ewe and sibling vocalisation [12], therefore, observing consistent results may become more difficult as litter size increases. Nevertheless, results were consistent between the paddock observations and the maternal-recognition test arena, where siblings were not present, indicating that the presence or absence of siblings was not influencing the results. Alternatively, it could be that the feeding treatments or BCS ranges were not extreme enough to consistently observe differences. Based on these findings, there is no advantage in terms of lamb bleating behaviour from *ad libitum* compared with pregnancy maintenance feeding, or from having ewes in BCS3 versus BCS2 in mid pregnancy.

Feeding treatment during late pregnancy did not influence any vigour behaviours of the lambs in the paddock or in the maternal-recognition test. This was consistent with the findings of Corner et al [17] and Gronqvist et al [15]. Furthermore, Everett-Hincks et al [21] reported no differences in vigour behaviours of lambs born to dams offered various pasture allowances in late pregnancy and through the lambing period and Kenyon et al [8,9] reported no advantage in total litter birth or weaning weight from feeding above pregnancy maintenance in late pregnancy. Combined these results indicate that for triplet bearing ewes, there is no advantage for their offspring to weaning from feeding at a level above pregnancy-maintenance requirements in late pregnancy.

Lamb behaviour in the maternal-recognition test was influenced by age of the lamb. Lambs tested at 24 h of age spent more time walking and reached the contact zone faster than lambs tested at 12 h. of age. This is consistent with previous studies, which showed older lambs were more mobile and faster or better at locating their mother [15,29]. This highlights the rapidity with which lamb behaviour develops over the first 24 h of life.

The inconsistencies in vocalisation among feeding treatments, combined with the similarities in MBS among feeding treatments indicate that *ad libitum* feeding of triplet-bearing ewes in late pregnancy is unlikely to be a reliable method of inducing improvements in the ewe behaviours examined here. Vigour behaviours and vocalisation of lambs were not improved by *ad libitum* feeding compared with feeding for pregnancy maintenance during late pregnancy, regardless of ewe body condition. Therefore, increased feeding during late pregnancy, above the level of pregnancy maintenance, in ewes within the present BCS range would not be effective as a management tool to improve behaviours of the ewe and her lambs soon after birth. Farmers need to consider other management options to improve ewe and lamb behaviour.

## Figures and Tables

**Table 1:** The effect of ewe feeding treatment and body condition score group (BCS 2.0, 2.5, or 3.0 at D72) on live weight and BCS of ewes (LS mean±SE)

Items	n	Live weight (kg)			Body condition score		
	
		D115	D136	D142	D115	D136	D142
Experiment one							
Feeding treatment							
Medium	61	77.6±0.8	86.2±1.5	90.7±1.5	2.64^b^±0.03	2.40^a^±0.06	2.51^a^±0.06
*Ad libitum*	58	77.7±0.8	89.3±1.4	92.0±1.5	2.52^a^±0.03	2.82^b^±0.06	2.72^b^±0.06
BCS group							
BCS 2.0	33	74.1^a^±1.1	85.7±1.9	89.2±2.0	2.08^a^±0.04	2.22^a^±0.08	2.09^a^±0.07
BCS 2.5	44	78.7^b^±1.0	89.8±1.7	94.1±1.7	2.68^b^±0.03	2.69^b^±0.07	2.72^b^±0.07
BSC 3.0	42	80.1^b^±1.0	87.8±1.7	90.8±1.8	3.09^c^±0.04	2.91^c^±0.07	2.94^c^±0.07

		**D114**	**D128**	**D142**	**D114**	**D128**	**D142**

Experiment two							
Feeding treatment							
Medium	34	76.1±0.8	81.4±0.9	89.1^a^±1.2	2.43±0.06	2.57±0.07	2.44±0.07
*Ad libitum*	37	75.7±0.8	80.7±1.0	97.1^b^±1.3	2.44±0.06	2.51±0.07	2.59±0.07
BCS group							
BCS 2.0	25	72.2^a^±1.0	78.2^a^±1.1	90.9^a^±1.5	2.18^a^±0.07	2.27^a^±0.08	2.33^a^±0.08
BCS 2.5	26	74.4^a^±1.0	79.8^a^±1.1	92.1^a^±1.5	2.33^a^±0.07	2.47^a^±0.08	2.39^a^±0.08
BSC 3.0	20	81.1^b^±1.1	85.3^b^±1.3	96.5^b^±1.7	2.80^b^±0.08	2.90^b^±0.09	2.84^b^±0.09

BCS, body condition score; LS, least squares; SE, standard error.

a–c Different superscripts within columns and main effects indicate values that significantly differ (p<0.05).

**Table 2:** The effect of ewe feeding treatment and body condition score group (BCS 2.0, BCS 2.5, and BCS 3.0 at D72) and their interactions on the percentage of lambs that emitted high- and low-pitched bleats and on the total number of high- and low-pitched bleats from each lamb in the paddock

Items	n	High-pitched bleat		Low-pitched bleat	
	
		Percentage	Number	Percentage	Number
Experiment one					
Feeding treatment					
Medium	183	91.6 (86.4–94.9)	17.9 (17.3–18.6)	57.2 (49.7–64.4)	2.4 (2.2–3.7)
*Ad libitum*	174	86.9 (81.2–91.1)	17.8 (17.2–18.5)	53.3 (46.0–60.5)	2.7 (2.4–2.9)
BCS group					
BCS 2.0	99	91.2 (83.8–95.4)	19.6^b^ (18.7–20.5)	53.6 (43.8–63.2)	2.4 (2.2–2.8)
BCS 2.5	132	91.0 (84.8–94.9)	20.3^b^ (19.5–21.1)	50.7 (42.2–59.1)	2.4 (2.2–2.7)
BSC 3.0	126	85.5 (78.2–90.6)	14.4^a^ (13.7–15.0)	61.3 (52.5–69.4)	2.7 (2.5–3.0)
Feeding×BCS1)					
Medium BCS 2.0	48	-	18.2^c^ (17.0–19.4)	-	3.0^cd^ (2.5–3.5)
Medium BCS2.5	69	-	20.8^d^ (19.7–21.9)	-	2.3^ab^ (2.0–2.7)
Medium BCS 3.0	57	-	15.2^b^ (14.2–16.2)	-	2.2^ab^ (1.8–2.6)
*Ad libitum* BCS 2.0	51	-	21.0^d^ (19.8–22.4)	-	2.0^a^ (1.6–2.4)
*Ad libitum* BCS 2.5	63	-	19.8^cd^ (18.7–20.9)	-	2.6^bc^ (2.2–3.1)
*Ad libitum* BCS 3.0	69	-	13.7^a^ (12.9–14.6)	-	3.2^d^ (2.8–3.7)
Experiment two					
Feeding treatment					
Medium	103	89.5^b^ (82.2–94.0)	17.4^b^ (12.9–14.4)	68.8 (59.1–77.1)	3.7 (3.3–4.1)
*Ad libitum*	110	78.4^a^ (69.1–85.5)	13.6^a^ (16.6–18.2)	70.5 (60.6–78.7)	3.5 (3.2–3.9)
BCS group					
BCS 2.0	75	71.2^a^ (59.8–80.6)	15.3 (14.4–16.2)	60.2^a^ (48.7–70.6)	2.1^a^ (1.8–2.5)
BCS 2.5	78	89.1^b^ (80.1–94.4)	14.4 (14.6–16.3)	64.5^a^ (50.4–71.6)	3.4^b^ (3.0–3.8)
BSC 3.0	60	89.4^b^ (79.0–95.0)	15.5 (14.6–16.6)	83.3^b^ (71.6–90.8)	6.6^c^ (6.0–7.3)
Feeding×BCS2)					
Medium BCS 2.0	45	-	16.5^bc^ (15.3–17.7)	-	2.6^b^ (2.2–3.1)
Medium BCS2.5	39	-	15.7^bc^ (14.5–17.0)	-	3.2^bc^ (2.6–3.8)
Medium BCS 3.0	27	-	21.3^d^ (19.6–23.1)	-	6.5^d^ (5.6–7.5)
*Ad libitum* BCS 2.0	30	-	14.1^b^ (12.9–15.5)	-	1.5^a^ (1.2–2.1)
*Ad libitum* BCS 2.5	39	-	15.4^bc^ (14.2–16.7)	-	3.6^c^ (3.0–4.2)
*Ad libitum* BCS 3.0	33	-	10.7^a^ (9.6–11.9)	-	6.6^d^ (5.8–7.6)

BCS, body condition score; CI, confidence interval.

1) Values are back-transformed means (95% CI).

2) Non-significant interactions are not presented.

a–e Different superscripts within experiments, columns and effects indicate values that significantly differ (p<0.05).

**Table 3:** The effect of the interaction of ewe feeding treatment and body condition score group (BCS 2.0, BCS 2.5 and BCS 3.0 at D72) on the number of high- and low-pitched bleats expressed by ewes that bleated at least once at the handling of their lambs1)

Items	n	High-pitched bleat	n	Low-pitched bleat
Experiment one				
Medium BCS 2.0	13	9.4^c^ (8.5–10.3)	14	18.0^c^ (16.8–19.2)
Medium BCS2.5	20	8.7^bc^ (8.0–9.4)	21	16.3^b^ (15.4–17.3)
Medium BCS 3.0	16	9.3^c^ (8.6–10.1)	18	10.6^a^ (9.8–11.5)
*Ad libitum* BCS 2.0	13	7.5^a^ (6.8–8.3)	15	18.7^c^ (17.6–20.0)
*Ad libitum* BCS 2.5	18	7.9^a^ (7.2–8.6)	18	19.0^c^ (17.9–20.1)
*Ad libitum* BCS 3.0	19	8.9^bc^ (8.2–9.6)	21	19.0^c^ (18.0–20.1)
Experiment two				
Medium BCS 2.0	12	6.5^b^ (5.7–7.3)	13	29.3^d^ (27.7–30.9)
Medium BCS2.5	9	6.4^b^ (5.6–7.2)	12	12.4^a^ (11.3–13.5)
Medium BCS 3.0	7	3.2^a^ (2.6–4.0)	9	25.8^c^ (23.9–27.8)
*Ad libitum* BCS 2.0	9	15.4^d^ (14.1–16.8)	9	20.5^b^ (18.9–22.1)
*Ad libitum* BCS 2.5	10	7.2^b^ (6.4–8.10	12	22.2^b^ (20.8–23.8)
*Ad libitum* BCS 3.0	10	12.8^c^ (11.7–14.1)	10	13.4^a^ (12.2–14.7)

BCS, body condition score; CI, confidence interval.

1) Values are back-transformed means (95% CI).

a–d Different superscripts within experiments, columns and effects indicate values that significantly differ (p<0.05).

**Table 4:** The effect of the interactions of ewe feeding treatment, body condition score group (BCS2.0, BCS2.5, and BCS3.0 at D72) and age at testing (12 h and 24 h) on the number of low- and high-pitched bleats expressed by lambs in the maternal recognition test

Items	n	High-pitched bleat	n	Low-pitched bleat
Feeding×BCS				
Medium BCS2.0	32	35.3^b^ (33.6–37.1)	44	8.0^d^ (7.3–8.9)
Medium BCS2.5	32	32.4^a^ (30.8–34.2)	44	4.5^b^ (3.9–5.1)
Medium BCS3.0	29	44.8^d^ (42.8–46.8)	71	8.4^d^ (7.5–9.3)
*Ad libitum* BCS2.0	25	39.5^c^ (37.6–41.4)	42	5.9^c^ (5.2–6.7)
*Ad libitum* BCS2.5	25	48.1^e^ (46.0–50.3)	41	3.8^a^ (3.3–4.5)
*Ad libitum* BCS3.0	29	41.0^c^ (39.2–42.9)	47	7.1^d^ (6.4–7.9)
Feeding×age				
Medium 12 h	54	41.4^b^ (40.0–42.9)	79	4.4^b^ (4.0–4.9)
*Ad libitum* 12 h	62	42.6^b^ (41.2–44.0)	89	3.3^a^ (2.9–3.7)
Medium 24 h	32	31.2^a^ (29.8–32.8)	54	10.7^c^ (9.9–11.6)
*Ad libitum* 24 h	25	43.0^b^ (41.1–45.1)	42	10.9^c^ (9.9–11.9)
Age×BCS group				
12 h BCS 2.0	35	41.6^b^ (40.0–43.3)	58	4.3^c^ (3.8–4.8)
12 h BCS 2.5	34	39.6^b^ (38.0–41.3)	56	3.5^a^ (3.1–4.0)
12 h BSC 3.0	35	45.0^c^ (43.2–46.8)	54	3.6^ab^ (3.1–4.1)
24 h BCS 2.0	20	28.7^a^ (26.8–30.7)	29	12.5^e^ (11.3–13.8)
24 h BCS 2.5	19	40.5^b^ (38.3–42.8)	29	5.4^d^ (4.7–6.3)
24 h BSC 3.0	25	39.4^b^ (37.4–41.5)	35	13.8^e^ (12.7–15.1)

BCS, body condition score.

a–f Different superscripts within experiments, columns and effects indicate values that significantly differ (p<0.05).
